# Utilizing a low-carbohydrate/high-protein diet to improve metabolic health in individuals with spinal cord injury (DISH): study protocol for a randomized controlled trial

**DOI:** 10.1186/s13063-019-3520-3

**Published:** 2019-07-30

**Authors:** Ceren Yarar-Fisher, Jia Li, Amie McLain, Barbara Gower, Robert Oster, Casey Morrow

**Affiliations:** 1 Department of Physical Medicine and Rehabilitation, UAB School of Medicine, 190 Spain Rehabilitation Center, 1717 6th Avenue South, Birmingham, AL 35233 USA; 2 Department of Nutrition Sciences, UAB School of Health Professions, 1675 University Blvd., Webb 624C, Birmingham, AL 35294 USA; 3 Department of Medicine/Division of Preventive Medicine, UAB School of Medicine, Medical Towers 642, 1717 11th Avenue South, Birmingham, AL 35205 USA; 4 Department of Cell, Developmental, and Integrative Biology, UAB School of Medicine, 1918 University Blvd, MCLM 680, Birmingham, AL 35233 USA

**Keywords:** Spinal cord injury, High-protein diet, Low-carbohydrate diet, Metabolic disease, Insulin sensitivity, Diabetes, Gut microbiome

## Abstract

**Background:**

Metabolic disorders (e.g., impaired glucose tolerance, insulin resistance, and type 2 diabetes) are more prevalent in people with spinal cord injury (SCI) than able-bodied individuals. Dietary modification is a more cost-effective treatment option than pharmacological therapies for reducing the risk of metabolic dysfunction. Lowering carbohydrate, increasing protein, and maintaining a proper dietary fat intake are expected to induce favorable adaptations in glucose control, body fat distribution, and the composition of the gut microbiome. However, dietary modification has not been rigorously investigated in people with SCI. The purpose of this study is to determine if an 8-week low-carbohydrate/high-protein (LC/HP) dietary intervention will show improvements in clinically important metrics of metabolic function, body composition, the composition of gut bacteria, and quality of life.

**Methods/design:**

We intend to recruit 100 participants with chronic traumatic SCI (3 years postinjury, C5–L2, American Spinal Injury Association impairment scale A–D, and aged 18–65 years) and insulin resistance, impaired glucose tolerance or untreated type 2 diabetes and randomly assign them to an 8-week LC/HP dietary intervention group or a control group. The daily LC/HP dietary intervention includes ~ 30% total energy as protein (1.6 g/kg per day) with a carbohydrate-to-protein ratio < 1.5 and fat intake set at ~ 30% of the total energy intake. The control group does not receive any dietary intervention and are continuing with their regular daily diets. Glucose tolerance, insulin sensitivity, β-cell function, body composition, gut microbiome composition, and quality of life measures are assessed at week 1, before starting the LC/HP dietary intervention, and at week 8, after completion of the LC/HP dietary intervention.

**Discussion:**

New information derived from this project will result in the development of a low-cost, simple, self-administered LC/HP dietary intervention for improving metabolic function in individuals with chronic SCI, improved understanding of the composition of gut bacteria in SCI, and how a LC/HP dietary intervention alters gut bacteria composition. In addition, this project will improve our understanding of the relationship between metabolic function and quality of life in individuals with long-standing SCI.

**Trial registration:**

ClinicalTrials.gov, NCT03207841. Registered on 5 June 2017.

**Electronic supplementary material:**

The online version of this article (10.1186/s13063-019-3520-3) contains supplementary material, which is available to authorized users.

## Background

More than 50% of individuals with spinal cord injury (SCI) are younger than 45 years of age [1, 2]. Therefore, more than half of individuals with newly acquired SCI have the potential to live a healthy and long life. While life expectancy has been increasing among individuals with SCI who survive the first year after injury, their life expectancy is still lower than that observed for the able-bodied US population [1, 3]. This is thought to result from secondary health conditions associated with neurological impairment, extreme physical inactivity, and deleterious body composition adaptations that ensue at the muscular [4], regional [5] and whole-body levels [6]. A significant decline in soft tissue lean mass and increased body fat mass has been repeatedly shown in the chronic stages of SCI [7, 8]. In addition, a positive relationship has been found between visceral adipose tissue cross-sectional area and fasting glucose and triglyceride levels [9]. Finally, completeness and higher level of injury have been shown to result in greater decline in lean mass compared with incomplete SCI [10, 11].

**Fig. 1:**
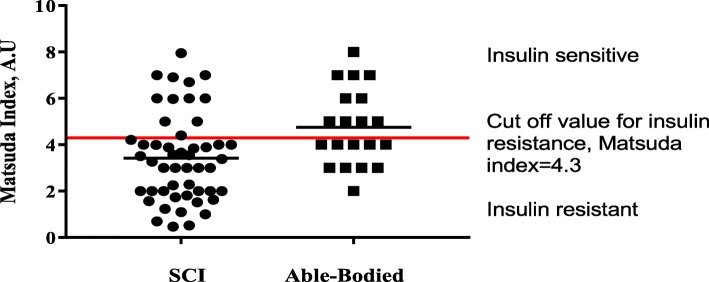
Distribution of insulin sensitivity estimated by the Matsuda index based on data obtained from the oral glucose tolerance test for individuals with spinal cord injury (SCI) versus able-bodied individuals. A.U arbitrary units

Secondary health conditions such as metabolic disorders (e.g., impaired glucose tolerance, insulin resistance, and type 2 diabetes) are more prevalent in people with SCI than able-bodied individuals [12, 13]. In fact, recent evidence from our laboratory shows a higher prevalence of insulin resistance among individuals with SCI (Fig. 1; ~ 80% in SCI patients versus ~ 50% in the able-bodied group, unpublished data). In addition, strong evidence [14–17] supports an SCI-specific threat for an accelerated trajectory of metabolic disorders, such that these health conditions occur at an earlier age in the SCI population than in able-bodied individuals, which often causes significant declines in functional independence, psychological health, and quality of life.

In addition to physical inactivity and changes in body composition at the muscular, regional, and whole-body levels, the composition and function of gut microbiome exert a significant role in the pathogenesis of metabolic dysfunction [18]. Fecal transplant studies in both human and animal models suggested a cause-effect relationship between gut microbiome and the development of insulin resistance [19, 20]. The human gastrointestinal tract is colonized by 100 trillion bacteria, the composition and function of which is susceptible to a plethora of environmental (diet, medication use, physical activity, etc) and host genetic factors. In the case of SCI, the loss of central nervous system control over the gastrointestinal tract, neurological bowel dysfunction and altered colonic transit time are implicated in disruption of the gut microbiota composition (i.e., gut dysbiosis (GD)) [21–24] . GD developing shortly after SCI is accompanied by inflammation of the intestine [22, 23], increased intestinal permeability (leaky gut), and bacterial translocation from the gut into distal organs [23]. Microbial products found in the circulation promote systemic inflammation and the development of metabolic impairment [25, 26]. Importantly, diet seems to strongly influence the bacterial composition of the gut [27]. For example, high-fat, high-carbohydrate diets (a Western diet) have been shown to increase Gram-negative gut bacteria, which triggers an immune response resulting in systemic inflammation that precedes insulin resistance and diabetes in humans [28]. Individuals with SCI, like those of the general US population, consume far more fat and carbohydrate than recommended levels [29], which may affect their gut microbiome composition and increase their risk of metabolic dysfunction.

**Fig. 2:**
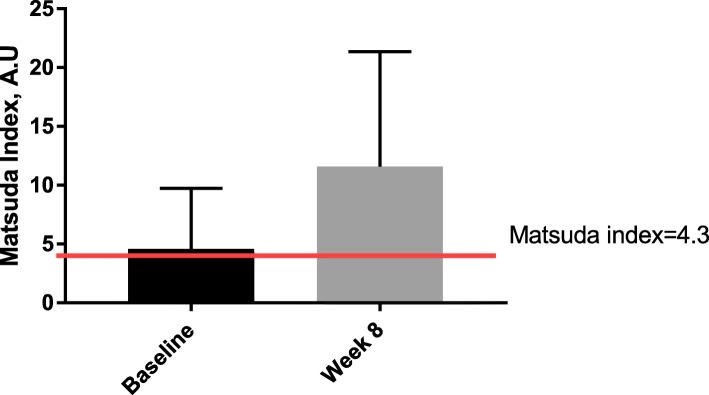
Whole body insulin sensitivity estimated using the Matsuda index based on data obtained from the oral glucose tolerance test. The red line indicates the suggested cut-off value for insulin resistance. Higher values indicate greater insulin sensitivity (i.e., less insulin resistance). Values are shown as means ± standard deviations. A.U arbitrary units

Dietary modification is an attractive countermeasure to the development of metabolic disorders in the SCI population. Lowering carbohydrate, increasing protein, and maintaining a proper dietary fat intake is expected to induce favorable adaptations in glucose control, fat distribution, and the composition of the gut microbiome. This improvement is thought to be due to the effects of protein on: 1) the sensation of feeling full after eating food despite similar or lower energy intake [30]; 2) contribution to storage of lean mass [31]; and 3) an insulin-sensitizing effect [32]. Our studies in SCI [33, 34] and other studies [35, 36] in able-bodied individuals with diabetes support this idea and demonstrate that it is possible to improve blood glucose control and insulin sensitivity in individuals with type 2 diabetes or impaired glucose tolerance by making relatively simple dietary adjustments, without the need for weight loss to improve glucose control. We have shown that increasing dietary protein from 15% to 30% of total food intake quantitatively increased insulin sensitivity from 4.6 (borderline insulin resistance) to 11.6 (insulin sensitive) in men with SCI who followed an 8-week low-carbohydrate/high-protein (LC/HP) dietary intervention (Fig. 2). Matsuda index values between four and five have been proposed as cut-off levels for insulin resistance among different able-bodied populations [37–40]. In addition, the LC/HP diet decreased homeostasis model assessment of insulin resistance (HOMA-IR) levels from 3.8 (insulin resistant) to 2.4 (insulin sensitive) [41]. These preliminary findings are both compelling and encouraging because extreme dieting (i.e., caloric deprivation) and weight loss were not required to achieve metabolic improvements in our study participants.

The experimental data in the SCI population are limited; therefore, it is critical to acquire a more detailed understanding of the effects of a LC/HP diet on metabolic function, body composition, and composition of the gut microbiome. Therefore, in this clinical trial, our purpose is to test the central hypothesis that individuals with SCI and with insulin resistance, untreated type 2 diabetes or impaired glucose tolerance who follow a LC/HP diet will show improvements in clinically important metrics of metabolic function (glucose tolerance, insulin sensitivity, β-cell function), body composition (increase in lean mass and decrease in total and visceral fat mass), the composition of gut bacteria, and quality of life.

## Research methods/design

### Study design and participants

**Fig. 3:**
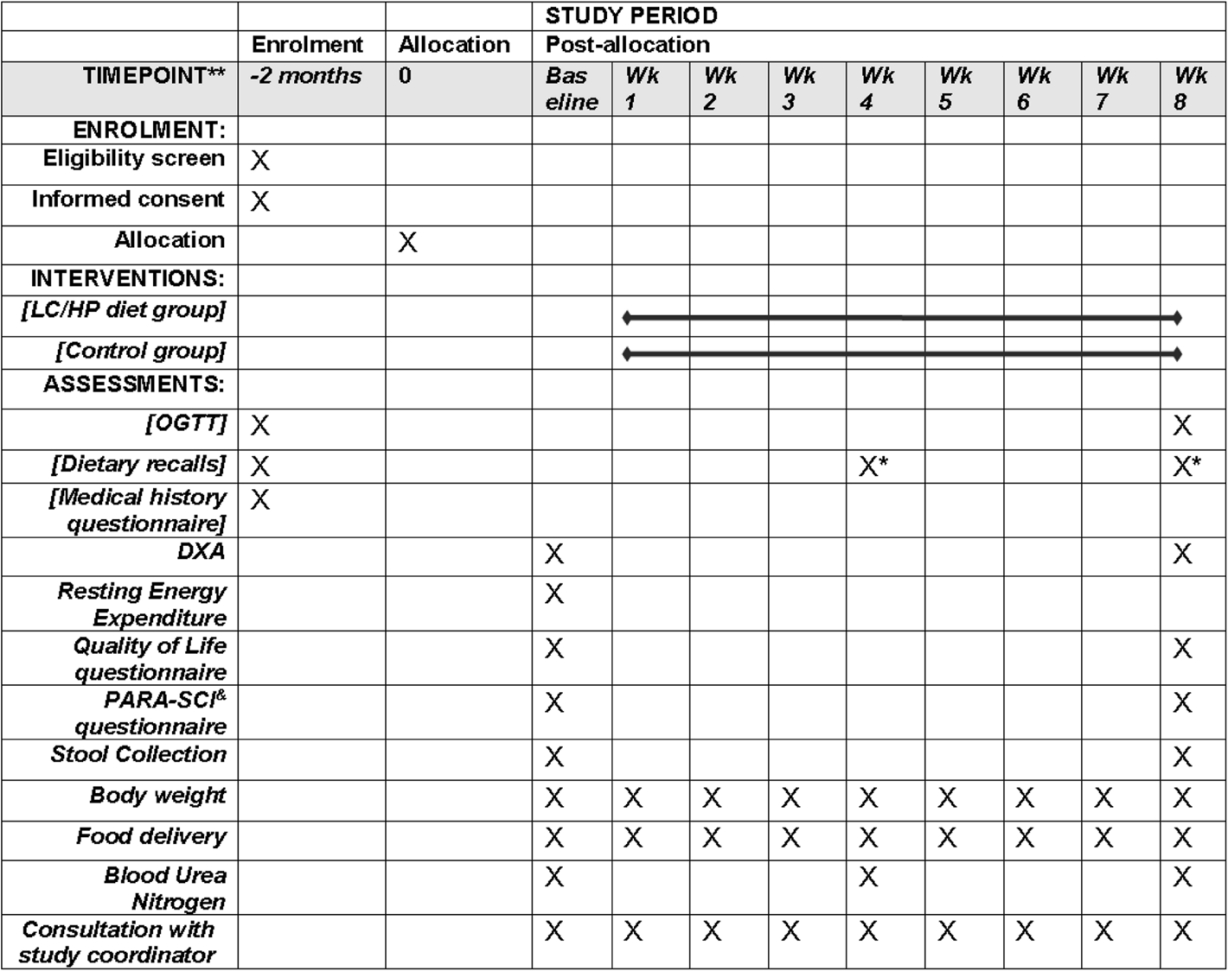
SPIRIT schedule of study enrolment, interventions, and assessments. *Dietary recall is only performed for the control group. ^*&*^Physical activity recall assessment for people with spinal cord injury

**Fig. 4:**
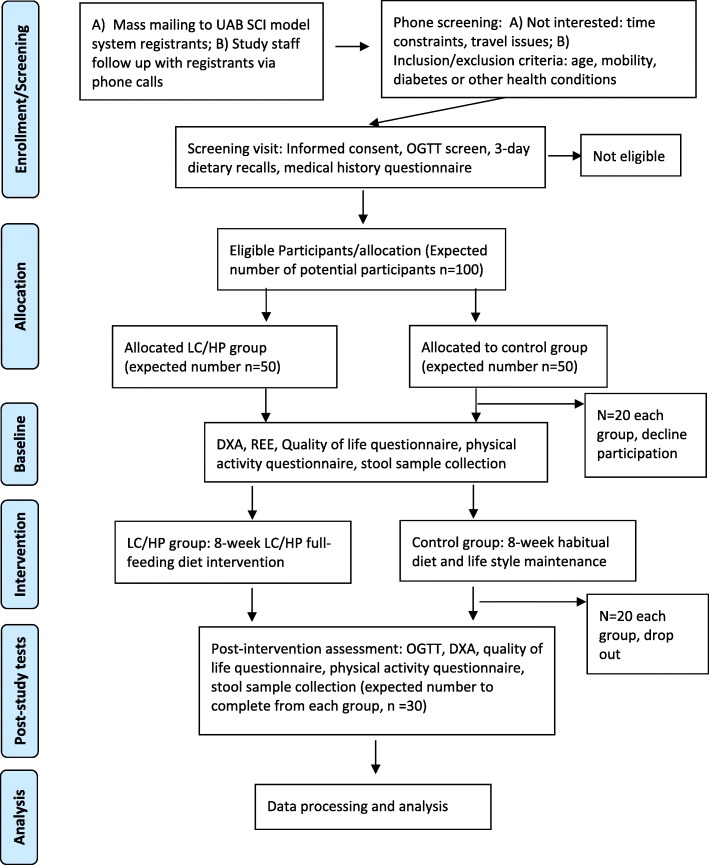
Study flow diagram. DXA: dual-energy x-ray absorptiometry, LC/HP: low-carbohydrate/high-protein, OGTT: oral glucose tolerance test, REE: resting energy expenditure, SCI: spinal cord injury, UAB: University of Alabama at Birmingham

In a single-masked, randomized controlled study design, eligible participants who provide informed consent are randomly assigned into a LC/HP dietary intervention group or a control group in a 1:1 ratio. Randomization is performed using the block randomization method (block size 4) by the study statistician (RO). A randomization list is generated, and assignments are placed into closed envelopes and given to each study participant by the study coordinator (JL, responsible for participant recruitment and trial coordination). All nurses performing blood draws, and core facilities analyzing the primary outcomes are blinded to group assignment. A Standard Protocol Items: Recommendations for Interventional Trials (SPIRIT) schedule is presented in Fig. 3 and the SPIRIT checklist is shown as Additional file 1. Outcome measures are performed at two time points: at baseline, before starting the LC/HP diet; and after completion of the 8-week LC/HP diet (pos-tstudy assessments are performed the morning after the last day of the intervention). A summary of data collection schedule for main outcomes is shown in Fig. 3, and the flow diagram for the overall study design is shown in Fig. 4. The study is currently being conducted at the University of Alabama at Birmingham (UAB) Center for Clinical and Translational Science (CCTS) Clinical Research Unit, UAB Diabetes Research Center Human Physiology Core, and UAB Nutrition and Obesity Research Center Metabolism Core, where participants in the intervention group are compensated $250 and participants in the control group are compensated $450 for their time. Participants are considered eligible for this study if they: 1) are between the ages of 18 and 65 years; 2) have a diagnosis of traumatic SCI at the cervical, thoracic, or lumbar level (C5–L2) classified as American Spinal Injury Association impairment scale (AIS) A, B, C or D; 3) impaired glucose tolerance or untreated type 2 diabetes; 4) no history of pre-existing self-reported type 2 diabetes and/or renal disease; and 5) are at least 3 years postinjury (in our preliminary studies we have demonstrated that glucose intolerance develops as soon as 3 years postinjury). Interested participants undergo an oral glucose tolerance test (OGTT) to confirm eligibility (type 2 diabetes, impaired glucose tolerance, or insulin resistance). We comply with the American Diabetes Association 2016 recommendations [42] to identify individuals with SCI with either: type 2 diabetes (2-h OGTT serum glucose ≥ 200 mg/dL) or impaired glucose tolerance/prediabetes (2-h OGTT serum glucose 140 mg/dL to 199 mg/dL). In addition, participants with Mastuda index below 4.3 are considered insulin resistant. Eligible participants are excluded if they develop new health conditions (e.g., pressure ulcers, kidney disease, heart disease, or need for hospitalization) that would affect study outcomes or inhibit them from participating [37].

The study population is expected to be representative for the demographics of the SCI patient population. Based on 2017 Spinal Cord Injury Facts and Figures, 81% of SCIs reported to the national database have occurred among males [2]. Therefore, we anticipate that among those who participate in this study, 81% will be male and 19% will be female. SCI is more common in non-Hispanic whites (63% non-Hispanic white, 22% non-Hispanic black, 11% Hispanic origin, 1% Native American, 2% Asian, and 1% other); therefore, we expect that more non-Hispanic whites, as compared with other racial or ethnic groups, will participate in this study. Children (aged < 18 years) are not eligible for this project.

### Recruitment

A two-stage procedure is employed to identify and recruit potential study participants. First, we use a computer-generated list of individuals with SCI who are enrolled in the UAB Spinal Cord Injury Model System (SCIMS). All potential participants are mailed a letter that describes the study, invites them to participate, and provides a return postcard indicating their willingness to participate. Responders are interviewed by telephone to assess eligibility. Three months after the mailing, participants who have not responded are called in a final attempt at recruitment. If the necessary sample size is not achieved, a second stage of recruitment begins, which utilizes advertising at the community level, such as at the Lakeshore Foundation, a not-for-profit organization that has been helping people with disabilities for more than 15 years by providing education, recreation, and other support services. The second stage of recruitment also includes advertising at the UAB SCIMS website, newsletters and social media. Those who respond to the advertisement are evaluated against the study eligibility criteria. Eligible responders are recruited consecutively as study participants until the necessary sample size is achieved. The recruitment and enrollment of participants are performed by the study coordinator (JL) or by study staff under study coordinator’s direct supervision.

Interested participants are invited for a screening visit. Study procedures, risks, and potential benefits are explained by the study coordinator (JL) or principle investigator (CY-F) during the screening visit. Participants’ rights are stated in the Institutional Review Board (IRB)-approved consent form. Participants are given options to provide consent for collection and use of their data and specimens in ancillary studies.

### Adequacy of the potential participant pool

Using the most conservative estimate reported in existing prevalence studies [12, 43], at least 12% (12–22%) of individuals with chronic SCI develop Type 2 diabetes. Based on our clinical research experience in the SCI population, approximately 60% of individuals with chronic SCI develop impaired glucose tolerance and insulin resistance. A total of 355 individuals with SCI who have had a follow-up visit with UAB SCIMS since January 2011 continue to live within the Greater Birmingham Metropolitan Area (1-h drive or less to our research facilities at UAB). We are planning 4 years for enrollment (allocating months 1–6 to project start-up and the last 6 months to data analysis); thus, we expect to have access to a pool of 355 individuals with SCI over the planned enrollment period. Applying the prevalence of 12% for type 2 diabetes and 60% for impaired glucose tolerance or insulin resistance in the SCI population, we estimate to have access to a total of 256 local potential participants with SCI. Based on our power calculations (described below), a sample size of 60 (30 per group) is needed to see a statistically significant effect of our dietary intervention. The available estimated sample pool of 256 local individuals with SCI should be adequate for our enrollment needs.

### Power calculations

Power calculations were performed using nQuery Advisor + nTerim 3.0 and assumed a two-sided statistical test and a significance level of 5%. We obtained estimates of the standard deviation (SD) for two clinically important metrics of metabolic function: a Matsuda index (indicator of insulin sensitivity) of 3.8 units [33], and 2-h OGTT serum glucose levels of 16 mg/dL [44]. Given the dearth of publications related to the effects of diet composition on gut bacteria, we were unable to perform power calculations for gut bacteria composition; however, we have adequate power to detect statistically significant differences in primary metabolic outcomes.

As described in Fig. 4, we intend to recruit 100 participants (50 per group) for the study, taking into account an estimated final participation rate of 80% and an attrition rate of 20%. With a final sample size of 30 participants per group (LC/HP diet and control), and also assuming a two-group *t* test and the prior assumptions, we have 80% power to detect between-group differences of 2.8 units in the Matsuda index, and 11.8 mg/dL in 2-h OGTT serum glucose levels as being statistically significant. With this same sample size, and also assuming a paired *t* test and the prior assumptions, we have 80% power to detect within-group differences of 2.1 units in the Matsuda index and 8.5 mg/dL in 2-h OGTT serum glucose levels as being statistically significant. We believe that these estimates are conservative because we will be performing our primary statistical analyses for between-group and within-group comparisons simultaneously using statistical methods that are more sophisticated than those that are assumed here.

### Interventions/groups

Consistent with the interest of the National Institute on Disability, Independent Living, and Rehabilitation Research (NIDILRR) in participatory action research, the first phase of the study involves participants in the personalization of their own menu plans consistent with the parameters of the LC/HP diet. In the second phase of the study, the personalized menus are tested in a randomized controlled fashion in individuals with SCI with insulin resistance, untreated type 2 diabetes, or impaired glucose tolerance.

#### LC/HP diet group

All LC/HP participants meet with our registered study dietitian to discuss their food- and nutrition-related history. Food-related history includes a participant’s usual dietary patterns (measured by three 24-h dietary recalls), favorite foods, any food allergies/intolerances, any difficulties with eating (limited hand function) or chewing or swallowing, and any nutritional concerns. The next step includes planning a personalized menu that is consistent with the LC/HP diet parameters (for the LC/HP diet group) based on participant-specific requirements identified during the meeting with the registered dietitian. During the first 2 weeks, participants try a six-day-roating menu and decide if they want to change/substitute any food item. The study dietician makes suggested changes at the beginning of the third week and the diet plan including six different menus remains the same for the rest of the study. The daily LC/HP diet includes ~ 30% total energy as protein (1.6 g/kg per day) with a carbohydrate-to-protein ratio < 1.5 and fat intake set at ~ 30% of the total energy intake. These dietary parameters are designed to fall within the Acceptable Macronutrient Distribution Range established by the Institute of Medicine [45]. The LC/HP dietary intervention meets the recommended daily intake for fiber, vitamins, and minerals for adults aged 18–60 years. Dietary fat sources focus on monounsaturated and polyunsaturated fats, e.g., plant oils and nuts; dietary carbohydrate sources emphasize whole grains, fruits, vegetables, and legumes; and dietary protein sources include lean meats, fish, chicken, eggs, and nonfat dairy foods, e.g., fat-free milk and low-fat cheese, consistent with American Diabetes Association and Institute of Medicine guidelines. Participants in the LC/HP can consume water, diet drinks, crystal light, and unsweetened tea/coffee without any cream, milk, or sugar. Two cheat meals have been incorporated into the diet plan. Participants can consume one "cheat meal" of their choice at weeks 3 (after the final menu is decided) and 6 (2 weeks before final testing). "Cheat day" food and drink consumption is recorded by our study team and included in our analysis.

All LC/HP meals are provided by the UAB CCTS Bionutrition Unit and are delivered to participants’ homes weekly. Every delivery includes breakfast, lunch, dinner, and snacks for 7 days (a 1-day sample menu is shown in Additional file 2: Table S1). Once a week during the study, participants are weighed at their home with a portable wheelchair scale (Health O Meter 2400KL, 800 lbs. capacity) to ensure weight stability. If weight changes exceed 2 kg from baseline, calorie modification is prescribed to maintain each participant’s weight.

The overall energy/calorie needs of each participant is determined according to resting energy expenditure (REE), assessed via indirect calorimetry and multiplied by an activity factor. The Physical Activity Recall Assessment for People with Spinal Cord Injury (PARA-SCI) [46] is used to determine physical activity level and an appropriate activity factor (for estimation of calorie needs) for each subject. The PARA-SCI is an interviewer-administered recall questionnaire tailored exclusively to people with SCI who use a wheelchair as their primary mode of locomotion. PARA-SCI is as reliable as measures that have been used in large-scale epidemiological studies in the general population [47]. Physical activity levels may be different among groups (control and intervention) during the course of the study, which may mix in with the effects of diet and distort the true relationship of the intervention to outcomes in the intervention group. Therefore, we measure each participant’s physical activity levels via PARA-SCI [46] and physical activity levels will be treated as a potential confounder in our statistical analyses.

#### Resting energy expenditure (REE)

REE is measured at baseline (week 1) after a 12-h fast. Measurements are performed in a quiet and softly lit room. Temperature is maintained between 22 °C and 24 °C. Participants lay supine on a comfortable bed, with the head enclosed in a Plexiglas canopy. After resting for 15 min, REE is measured for 30 min with a computerized, open-circuit, indirect calorimetry system with a ventilated canopy (Vmax ENCORE 29 N Systems, SensorMedics Corporation, Yorba Linda, CA). The last 20 min of measurement is used for analysis. O_2_ uptake and CO_2_ production is measured continuously and values are averaged at 1-min intervals. REE is calculated from the O_2_ and CO_2_ data.

#### Safety of the LC/HP dietary intervention

Administering a LC/HP diet for a relatively short period of time (8 weeks) is not expected to produce any significant side effects. There have been no reported adverse side effects of this diet in long-term or short-term clinical trials in healthy individuals or patients with diabetes [48–50]. In fact, studies involving administration of a LC/HP diet for a relatively longer period of time (8–16 weeks) in obese patients and/or patients with diabetes have shown improvements in metabolic and cardiovascular disease markers (visceral fat, blood glucose, and insulin levels) [51, 52].

#### Retention and compliance

We expect voluntary attrition to be comparable to that of our diet and exercise training trials in the SCI population (20%). Participants who voluntarily withdraw prior to completing 8 weeks of the study or data collection will be replaced with a new recruit on a per-participant basis. Ensuring compliance with home consumption of the prescribed LC/HP diet may be challenging. In a prior study involving a LC/HP diet in individuals with SCI, CY-F (the Principal Investigator (PI)) demonstrated nearly 100% compliance [33]. In any event, subjects are given preconfigured meals with instructions to consume all prescribed foods. Dietary compliance is maximized via education with study coordinator, as well as involvement of the participant in individual food item selection from a menu of options (personalized menus). To improve both dietary and study retention, subjects assigned to the LC/HP diet group are contacted by telephone 2 days per week to troubleshoot any potential barriers (food craving, poor appetite, constipation, loose or firm stool, and so on) to their progress in the study by the study coordinator (JL). A daily food checklist is provided to record adherence to the prescribed menu. Blood urea nitrogen (BUN) is measured before, at week 4, and at week 8 as a surrogate indicator of dietary protein intake during the study [53, 54].

#### Control group

The control group does not receive any dietary intervention and are continuing with their regular daily diets; however, initial meetings with a registered dietician are required to gather information about their food- and nutrition-related history and to identify any control group participants who are already committed to a LC/HP diet. Individuals who are already following a LC/HP diet plan are not eligible to participate. Participants in the control group complete three 24-h food recalls (on two weekdays and one day at the weekend) three times (at weeks 1, 4 and 8) during the course of the study to gather dietary information including dietary intake and/or particular aspects of the diet. Participants are asked to recall foods and beverages they consumed in the 24 h prior to the interview. Three 24-h food recalls appear optimal for estimating energy intake [55, 56].

### Outcome measures and analysis

#### Primary outcomes

The primary outcomes are as follows: 1) metabolic function (glucose tolerance, insulin sensitivity, β-cell function and lipid profile); 2) body composition (total fat mass, total lean mass and visceral fat mass)l; and 3) gut microbiome (composition of gut bacteria).

#### Clinical procedures to measure metabolic function

For the OGTT, each subject consumes a 75-g oral glucose load within 5 min. Blood samples are collected immediately before and 10, 30, 60, 90 and 120 min after glucose ingestion for measurement of serum glucose and insulin. Blood for serum glucose determination is collected with sodium fluoride and blood for serum insulin is collected with heparin. Assays are performed in the UAB Human Physiology and Metabolism Core. Serum glucose, insulin, and C-peptide values are analyzed for measures of insulin sensitivity and β-cell (cells that control insulin secretion in the pancreas) function using mathematical modeling techniques developed for data generated from the OGTT [52]. Whole body insulin sensitivity (WBIS) is also calculated using the Matsuda index (a formula based on insulin and glucose values measured during the OGTT) [57]. The HOMA-IR is calculated using the following formula: HOMA-IR = $\frac{\mathrm{glucose}\ \left(\mathrm{mg}/\mathrm{dL}\right)\times \mathrm{insulin}\ \left(\upmu \mathrm{U}/\mathrm{mL}\right)}{405}$ [58]. Serum glucose and C-peptide assays are performed on an automated analyzer (Sirrus analyzer; Stanbio Laboratory, Boerne, TX) and serum insulin is measured using an immunofluorescent method with an AIA-600 II analyzer (TOSOH Bioscience, South San Francisco, CA) per the manufacturers’ instructions.

For lipid analysis, venous blood samples are collected to measure the participant’s total cholesterol, triglycerides (TG), high-density lipoprotein cholesterol (HDL-C), and low-density lipoprotein cholesterol (LDL-C) levels in the fasting state. This blood is drawn using the catheter that is placed for the OGTT so it does not require an extra needle stick. Total cholesterol, HDL-C, and TG are measured with the Sirrus analyzer. LDL-C levels are estimated using the formula LDL-C = total cholesterol − HDL-C − triacylglycerol/5 [59]. Analyses are performed in the UAB Human Physiology and Metabolism Core.

#### Clinical procedures to measure body composition

We use dual-energy x-ray absorptiometry (DXA) to measure body composition. Total body imaging (to measure total fat and lean mass and visceral fat mass) is acquired using the GE Healthcare Lunar iDXA (GE Healthcare Lunar, platform version 16, Madison, WI) and analyzed using enCORE software version 13.6 in the UAB Human Physiology and Metabolism Core. Daily quality-control scans are acquired during the study period. Participants are scanned using our standard imaging and positioning protocols used both in able-bodied individuals [60, 61] and the SCI population [62] . Participants are placed on the center of DXA scanner in a supine position with the arms facing medially thumbs-up. The legs are strapped to secure the knees and feet to prevent movement during the scan. For measuring visceral fat, a region of interest is automatically defined, with the caudal limit placed at the top of the iliac crest and the height set to 20% of the distance from the top of the iliac crest to the base of the skull to define the cephalad limit. Fat mass data from DXA are transformed into CT adipose tissue volume using a constant correction factor (0.94 g/cm^3^) [63]. This constant is generally consistent with the density of adipose tissue.

#### Procedures to measure changes in the composition of gut bacteria

Gut bacterial (microbial) composition patterns are determined from stool samples using our established protocol [64]. Stool collection occurs at participants’ homes. Participants or their caregivers are provided with a prepaid and labeled FedEx package containing a stool collection kit (Para-pak vial for sample preservation, stool collection container, 2-gal Ziploc bag for disposal of collection container, and sanitizing wipes). After bowel movement, approximately 5–10 mL of stool is transferred to the Para-pak vial using the attached spatula. For participants who have their bowel management program on their beds, the stool collection container is not used. Fedex packages containing stool samples are shipped to our laboratory using the overnight shipping option to ensure appropriate preservation of the microbiota. Upon delivery, the fecal samples are diluted in Cary-Blair medium to 0.1 mg/mL for a total volume of 20 mL with 10% by volume glycerol. Aliquots of 200 μL are stored at −80 °C for DNA extraction/gut bacteria analysis. Fecal DNA is isolated via a Fecal DNA Isolation Kit. The 16S rRNA gene amplification protocol with the MiSeq System is used to characterize the composition of gut bacteria to the family level, and in some cases the genus and species level [64]. The relative abundance of identified organisms is normalized prior to analysis based on maximum read counts per sample. The organisms with low relative frequencies (< 0.1%) are filtered. The remaining organisms are used for within-subject and between-subject comparison analyses.

#### Secondary outcome

The secondary outcome is health-related quality of life (QOL). This is measured via the SCI-QOL measurement system, which consists of a set of items that have been developed specifically for use in spinal cord medicine [65, 66]. Two scales that measure physical and health-related QOL are being used (Table 1).

**Table 1:** List of spinal cord injury quality of life (SCI-QOL) questionnaire domains and items used in the study

Physical–medical health	
Pressure ulcers	
Bladder management difficulties	
Bladder complications	
Bowel management difficulties	
Pain interference	
Pain behavior	
Physical function	
Basic mobility	
Ambulation	
Fine motor	
Self-care	
Wheelchair mobility	

### Statistical analysis

All data analyses will be carried out using data from participants that complete the study intervention. Descriptive statistics will be calculated for all study parameters of interest. Analyses for all aims include comparisons of means of outcome measures, which include the Matsuda index (insulin sensitivity), 2-h OGTT serum glucose levels (glucose tolerance), β-cell function, total cholesterol, TG, LDL-C, and HDL-C, total lean mass, total fat mass, visceral fat mass, and composition of gut bacteria between the two groups (LC/HP diet and control diet) as well as comparisons within each group of the changes from the baseline visit to the week 8 visit. The primary method of analysis is mixed models repeated measures analyses, such as repeated measures analysis of covariance. An appropriate structure for the covariance matrix (e.g., the unstructured covariance matrix) will be selected for these models using the final data. This method allows us to compare changes over time (8 weeks) and differences between groups simultaneously. Potential confounders, such as age, gender, injury level, time since injury, and levels of physical activity, will be accounted for in these analyses. Overall adjusted comparisons between groups at baseline will be performed using the two-group *t* test, and overall unadjusted comparisons of the change from the baseline visit to the week 8 visit is performed using the paired *t* test. If assumptions of normality of distribution for the above tests are not tenable, variables may be log10 transformed prior to analysis or appropriate nonparametric tests such as the Wilcoxon rank-sum and signed-rank tests may be used. Generalized linear model techniques, such as regression analyses and mixed models repeated measures analyses, will be used to determine how changes in composition of gut bacteria are associated with improvements in metabolic function, or how improvements in metabolic function are related to improvements in quality of life. Secondary analysis of participants who have type 2 diabetes or glucose intolerance will be performed if the numbers of participants with these conditions differ greatly between the study groups. If we have missing data at study completion, we will not perform multiple imputation or apply the last observation carried forward method; instead, we will perform a secondary analysis of participants who completed the study versus participants who did not complete the study and determine if these two groups differ in any substantial way. Statistical tests will be two-sided and performed using a 5% significance level. SAS software, version 9.4 or later, will be used to conduct the statistical analyses.

### Data management

The Data and Safety Monitoring Committee (DSMC) includes the study coordinator and principle investigator. In addition, a data management plan has been created. All study data are managed in a central database using REDCap, a secure web-based software system designed for clinical trials. All questionnaires are coded into REDCap and administered electronically. Data from outcome measures are output from the respective technical equipment in electronic or paper form and then uploaded directly into the REDCap database. However, data on adverse events that are collected by the research team are recorded on paper forms, stored in the Clinical Research Unit, and then manually entered into the REDCap database. Food intake data are captured on paper forms and double entered into the database.

Paper documents are scanned and saved on UAB’s secure network and/or stored in locked cabinets in the research coordinator’s office. Data stored on UAB’s secure network are protected through stringent security measures assured by UAB’s technical department and through the use of coded ID numbers and electronic security systems required by the Health Insurance Portability and Accountability Act (HIPAA) of 1996. The REDCap software system is hosted locally on secure servers provided by the UAB School of Medicine. REDCap software leaves a pristine audit trail by documenting all changes to data, logging all user activity, and recording all pages viewed by every user. Moreover, it strictly controls access to data with password authentication and user privileges. This ensures that all users have limited access to only the data and information that they need or to which they should have access.

All data that are captured on paper forms and then manually entered into the database are verified by double entry (enter/verify). The REDCap database has logic checks built in to identify valid values, and any potentially erroneous data will be meticulously documented and triple entered. The PI and study coordinator will periodically check the database for missing data and will document all such data and the reasons for absence. After the study is completed, all data will be quality checked by the statistician, PI, and the study coordinator in year 5; tasks will be divided among the three personnel according to their respective responsibilities. Statistical analyses for each endpoint will begin only after all data for that endpoint has been quality checked. In compliance with UAB’s IRB policies, all data and records will be kept for at least 3 years after the study is completed; all protected health information (PHI) for participants will be deleted 3 years after the trial is completed, while the deidentified final dataset will be retained indefinitely and published online for the benefit of other scientists.

### Trial monitoring

No regular external trial auditing is scheduled. However, all human participant data, ranging from recruitment/screening to diet intervention and testing to laboratory tests, are reviewed in quarterly (every 3 months) in DSMC meetings attended by key study staff and the PI. This includes updates on any new hazards, risks, or adverse events, and plans of action. Additional investigators and staff will be asked to participate as the need for their input or expertise arises. If during the course of these meetings particular unforeseen hazards or risks are identified that may predispose patients to an unusually high number of serious adverse events, the PI will consult the appropriate members of the investigative team, as well as the UAB IRB, to determine if the study should be terminated or altered in some way. Any procedure that is deemed hazardous will be eliminated from the study and replaced with an alternative if one with reasonable risk can be identified.

### Adverse event monitoring and reporting

Participants are instructed to report adverse events as they occur. In case of emergency, participants are given a telephone number to enable them to contact the study physician. Participants are given a separate telephone number and email address to contact the study team to report nonurgent adverse events.

#### Documentation

All adverse events will be documented on specialized case report forms and graded on their attribution (unrelated to the protocol, or possibly, probably, or definitely related to the protocol), severity (mild, moderate, or severe), expectedness (unexpected versus expected), and frequency. We will also document any actions taken related to the adverse event and the outcome or resolution of the event.

#### Reporting

All serious or unexpected adverse events will be reported to the UAB IRB with a description of the event, when and how it was reported, and appropriate documentation to corroborate the event. The description of the adverse event will include all information listed on the case report forms. The IRB will then determine whether additional reporting to the NIDILRR is required. All adverse events will be reported immediately to both the study physician and PI, and then, in turn, reported to the IRB within 10 business days.

### Protocol amendments

Any change to the protocol will require a written protocol amendment that must be approved by NIDILRR and IRB before implementation. Upon acceptance from the sponsor and IRB, the PI will make updates and edits to the study record published on ClinicalTrials.gov. If the PI determines that an immediate change to or deviation from the protocol is necessary for safety reasons to eliminate an immediate hazard to the subjects, the IRB will be notified immediately.

### Confidentiality

To protect privacy, each study participant is assigned a unique three-digit identification number that cannot be traced to any PHI. The PI and study coordinator code all data forms, participant information, and biological specimens using these ID numbers. No PHI appears on these materials; instead, the keys linking participants’ identities to their unique identification numbers are being stored separately on a passcode-secured storage disk. To protect privacy and confidentiality, any original paperwork documenting a participant’s name and PHI are stored in a locked cabinet in the research coordinator’s office, while any digital study records involving PHI are stored in REDCap and/or on computers requiring password authentication that are stored in locked offices and that are behind secure firewalls. Paperwork involving PHI are kept to the bare minimum necessary and are stored in a locked cabinet in the research coordinator’s office. Thereafter, each generated data and specimen are labeled with a unique anonymous ID number.

### Access to data

Only study staff, the UAB IRB, official overseers of clinical research at UAB, and representatives of the NIDILRR have access to study records, data, and specimens; all access is on a need-to-know basis. Study staff include all key personnel, the dietician, the study coordinator, the study statistician, and any other individuals who perform specific tests or procedures for the study, such as nurses and laboratory technicians. All study staff are trained in HIPAA standards for protecting PHI and will not refer to PHI or confidential information in the presence of individuals outside of the study team. Moreover, each study staff member’s access to participants’ data is limited to only the functions for which they are responsible. Records that identify study participants are kept confidential as required by law, and every effort is made to maintain the confidentiality of participants’ study records. Except when required by law or if necessary to protect their rights or welfare, study participants are not identified by name or any other identifying characteristic in records disclosed to those outside of the study staff.

### Dissemination policy

We will disseminate our results in several ways. First, we will publish our results in peer-reviewed journals with open-access policies and/or in top-tier journals. Second, we will present our results at scientific conferences, including at the American Congress of Rehabilitation Medicine annual meeting, the American Spinal Injury Association annual meeting, and the Experimental Biology annual meeting. Third, using electronic media, we will publish our findings on the UAB Spinal Cord Injury Model System Information Network website. This website features a comprehensive collection of links (over 360 to date) to SCI-related information provided by reputable organizations, associations and educational institutions. All educational materials written and produced from this project will be made available free on this website. We will also use the “Pushin’on” electronic newsletter of the UAB SCIMS*.* This newsletter provides persons with SCI and their families with information of interest. Over the years, the newsletter has featured original articles along with news, information, and synopses of research of importance to individuals with SCI. A synopsis of the proposed research trial, including a description of the diet intervention and a lay summary of major findings and how to implement them will be published on the newsletter.

Fourth, we will use the Nutritional Education Initiative. We have designed a comprehensive strategy to promote awareness, education, and utilization of information on proper nutrition. First, we will collaborate with the Model System Knowledge Translation Center to develop a fact sheet on nutrition after SCI. Second, we will develop a cookbook that will offer recipes, cooking directions and ingredient shopping lists for nutritionally sound meals that are recommended and approved by our consulting dietitian. Finally, we will develop nutrition videos and an accompanying cookbook “app” in collaboration with the National Center on Health, Physical Activity and Disability, which is a knowledge translation and dissemination branch of the UAB/Lakeshore Research Collaborative.

## Discussion

Feasible interventions to improve metabolic function in the chronic SCI population are in great demand. Given that metabolic disorders severely compromise health outcomes and important domains of quality of life, including participation in daily life and community living and employment outcomes, targeted strategies to combat these morbidities are of paramount importance. Compared with pharmacologic therapies, dietary modification is a more cost-effective treatment option for reducing the risk of metabolic dysfunction that, surprisingly, has not been rigorously investigated in people with SCI. Much like the general US population, individuals with SCI consume far more fat and carbohydrate than recommended levels [29]. High-fat, high-carbohydrate diets (Western diet) have been associated with a higher prevalence of obesity and metabolic disease [67–69] as well as negative adaptations in the diversity of gut bacteria (increased levels of harmful bacteria and reduced levels of beneficial bacteria) that usually precedes the development of diabetes or insulin resistance [28]. Therefore, macronutrient (carbohydrate, protein, and fat) modification may be able to prevent or correct the impaired metabolic state in the SCI population. Thus, results from our study will be the first to determine if 8 weeks of a LC/HP diet (versus control) will improve glucose tolerance, insulin sensitivity, β-cell response, body composition, diversity of gut bacteria and decrease total cholesterol, TG, LDL-C. There is a paucity of research [24] on gut bacteria (microbiome) within the SCI population; therefore, our study will be among the first to generate preliminary gut bacteria profiles of individuals with SCI who have metabolic disorders and receive a LC/HP diet.

New and insightful information to be derived from this project will include: 1) development of a low-cost, simple, self-administered LC/HP dietary intervention for improving metabolic function in individuals with chronic SCI; 2) demonstration of the efficacy of a LC/HP diet as a nonpharmacological option for improving blood glucose control, insulin sensitivity, β-cell function, and whole body and regional body composition; 3) improved understanding of the composition of gut bacteria in SCI and how a LC/HP dietary intervention alters gut bacteria composition; 4) increased knowledge of the influence of gut bacteria on metabolic function in SCI; and 5) improved understanding of the relationship between metabolic function and quality of life in individuals with long-standing SCI.

In conclusion, there are several innovative features of the proposed experiments that are expected to significantly advance the field and improve metabolic function, with the overall goal of reducing metabolic disease risk among individuals with long-standing SCI. A dietary intervention that results in improvements in metabolic function can be implemented anywhere in the world at low cost and without major regulatory hurdles. Better metabolic function in individuals with SCI will contribute to a significantly higher quality of life as well as improved long-term health outcomes.

## Trial status

The study has been active and open for enrollment since September 2017. The first patient was enrolled on 1 November 2017, and 23 patients were enrolled as of January 2019. Enrollment is expected to be completed in September 2021. The protocol version is version H, 21 December 2018.

## Additional files

Additional file 1: SPIRIT checklist. (DOC 128 kb)

Additional file 2: **Table S1.** Sample study menu. (DOCX 13 kb)

## Data Availability

The datasets used and/or analyzed during the current study will be available from the corresponding author on reasonable request.

## Abbreviations

AIS: American Spinal Injury Association (ASIA) impairment scale
BUN: Blood urea nitrogen
CCTS: Center for Clinical and Translational Science
DSMC: Data and Safety Monitoring Committee
DXA: Dual-energy x-ray absorptiometry
GD: Gut dysbiosis
HDL-C: High-density lipoprotein cholesterol
HIPAA: Health Insurance Portability and Accountability Act (1996)
HOMA-IR: Homeostasis model assessment of insulin resistance
IRB: Institutional Review Board
LC/HP: Low-carbohydrate/high-protein
LDL-C: Low-density lipoprotein cholesterol
NIDILRR: National Institute on Disability, Independent Living, and Rehabilitation Research
OGTT: Oral glucose tolerance test
PARA-SCI: Physical Activity Recall Assessment for People with Spinal Cord Injury
PHI: Protected health information
PI: Principal Investigator
QOL: Quality of life
REE: Resting energy expenditure
SCI: Spinal cord injury
SCIMS: Spinal Cord Injury Model System
SD: Standard deviation
SPIRIT: Standard Protocol Items: Recommendations for Interventional Trials
TG: Triglycerides
UAB: The University of Alabama at Birmingham
WBIS: Whole body insulin sensitivity
