# Supplementary material for: Utilizing a low-carbohydrate/high-protein diet to improve metabolic health in individuals with spinal cord injury (DISH): study protocol for a randomized controlled trial
Source: Trials. 2019 Jul 30;20:466. doi: 10.1186/s13063-019-3520-3 (PMC6664761; doi:10.1186/s13063-019-3520-3)
Supplement: Supplementary file 2 — Table S1. Sample study menu. (DOCX 13 kb) [file 13063_2019_3520_MOESM2_ESM.docx]

|  | **Day 1 Meal/Food** | **Amount** |
| --- | --- | --- |
| **Breakfast** | Boiled egg | 1 large |
|  | Wheat bread | 2 slices |
|  | Promise margarine | 10 g |
|  | Jelly, regular | 2 pkts |
|  | Turkey bacon | 3 slices |
|  | Mandarin oranges, juice packed | 8 oz |
| **Lunch** | Whole wheat tortilla | 2 (8 in) tortillas |
|  | Deli turkey | 150 g |
|  | Kraft 2% American cheese | 2 slices |
|  | Mustard | 2 pkg |
|  | Pretzels | 1 (0.9 oz) bag |
|  | Applesauce, unsweetened | 8 oz |
| **Dinner** | Chicken breast, baked | 180 g |
|  | Broccoli, frozen | 1 cup |
|  | Promise margarine | 5 g |
|  | Lettuce | 1/8 head wedge |
|  | Kraft Italian dressing, regular | 2 (7/16 oz) pkgs |
|  | Skim milk | 8 FO |
|  | SF Swiss Miss hot cocoa mix | 1 pkg |
| **ADD-IN** | Multi-grain Cheerios mix | 45 g |
|  | Isopure powder | 18 g |
|  |  |  |
|  | **Day 2 Meal/Food** | **Amount** |
| **Breakfast** | Pillsbury Scrambles: Cheese egg & bacon | 1 each |
|  | Breakfast sausage, meatless | 1 links |
|  | Orange, fresh | 1 medium |
|  | Skim milk | 8 FO |
| **Lunch** | LC Chicken Fettuccini | 1 pkg |
|  | Spinach, canned | 8 oz |
|  | Promise margarine | 5 g |
|  | Peanuts, regular | 50 g |
|  | 100 kcal fruit cocktail | 8 oz |
| **Dinner** | Tilapia | 8 oz (2 filets) |
|  | Broccoli, cauliflower & carrot mix, frozen | 1 cup |
|  | Green peas, canned | 8 oz |
|  | Promise 60% spread margarine | 10 g |
|  | English muffin, whole wheat | 1 whole |
|  | Light cream cheese | 1 oz |
| **ADD-IN** | Almond mix | 15 g |
|  | Protein bar | 1 bar |

Table S1. Sample study menu
